# Supplementary material for: A pilot-scale comparison between single and double-digest RAD markers generated using GBS strategy in sesame (Sesamum indicum L.)
Source: PLoS One. 2023 Jun 2;18(6):e0286599. doi: 10.1371/journal.pone.0286599 (PMC10237379; doi:10.1371/journal.pone.0286599)
Supplement: S1 File — (DOCX) [file pone.0286599.s001.docx]

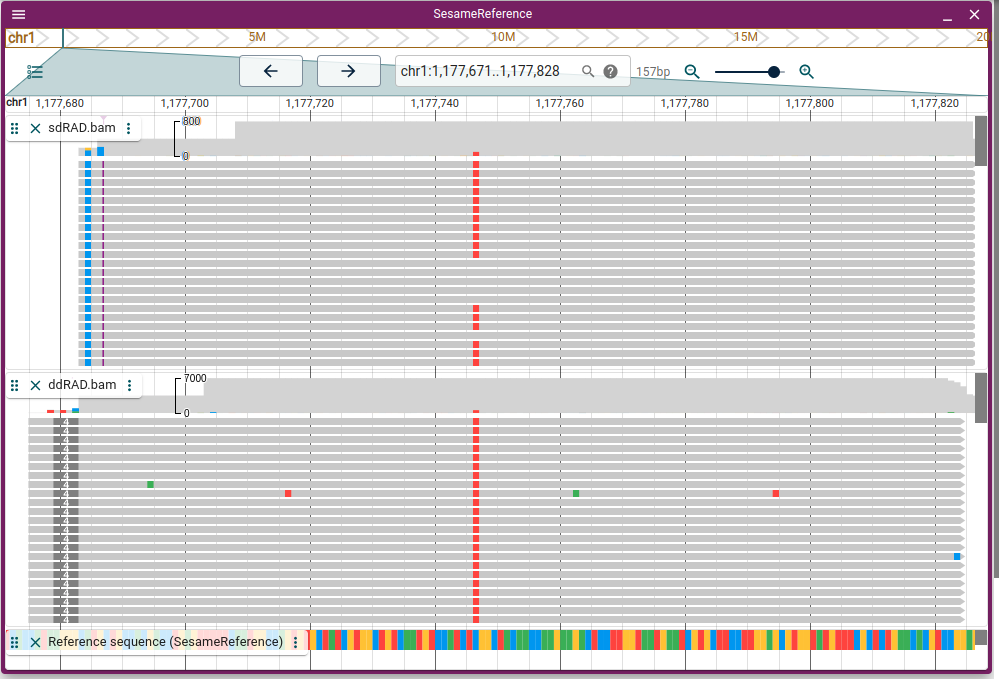


**Supplementary Figure 1:** Jbrowse2 display of the common SNPs between sdRAD-seq and ddRAD-seq datasets.


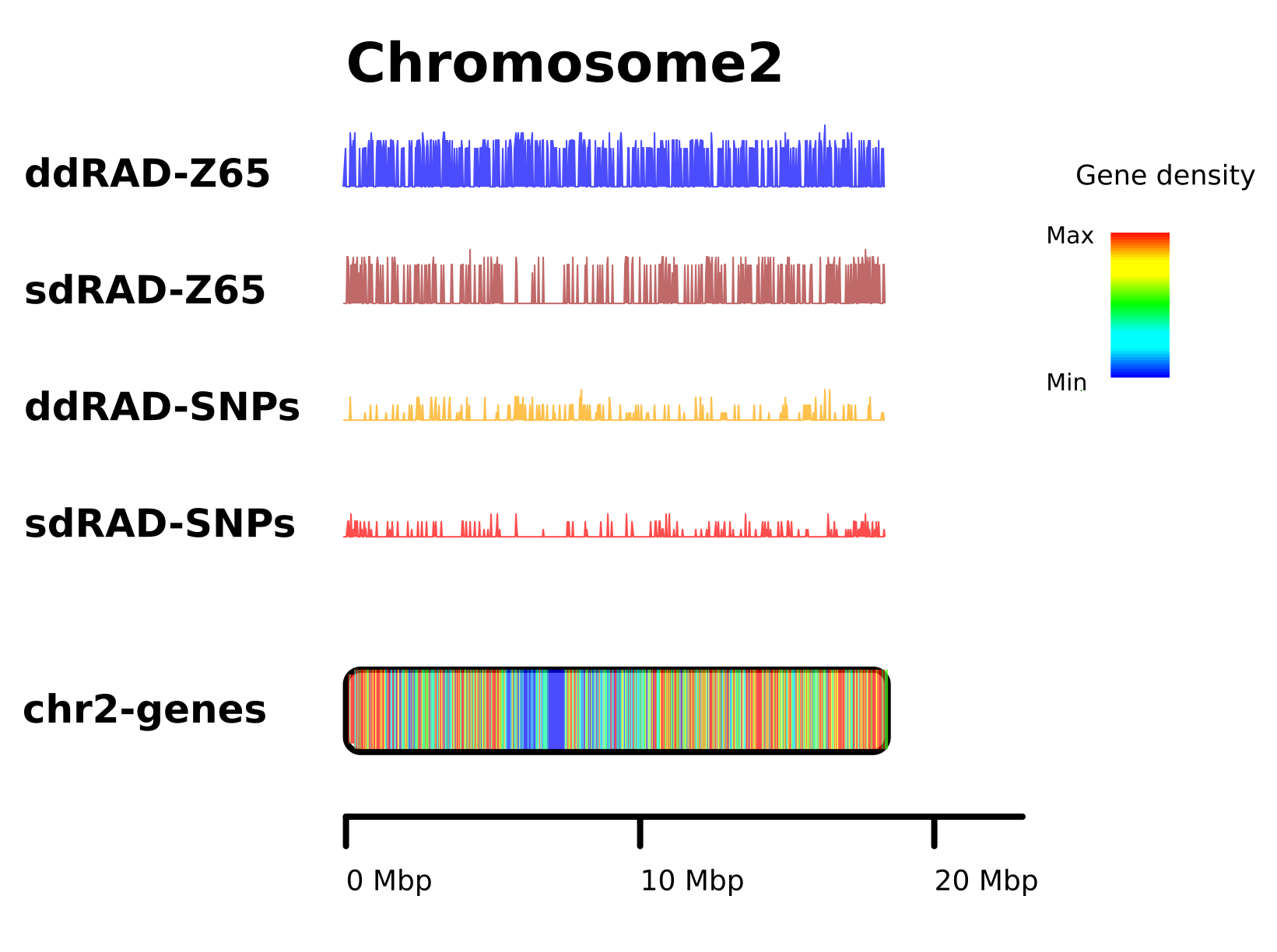

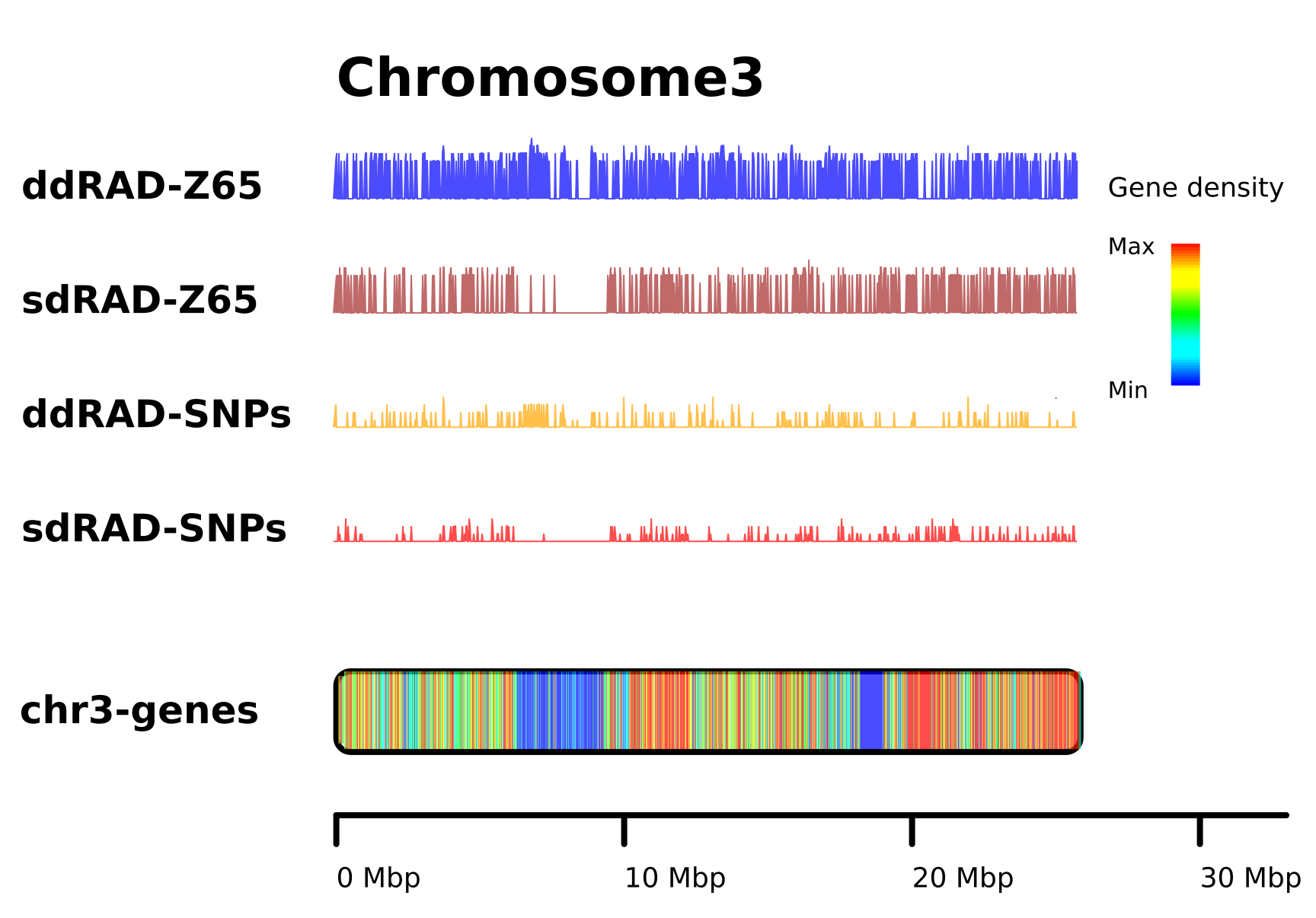

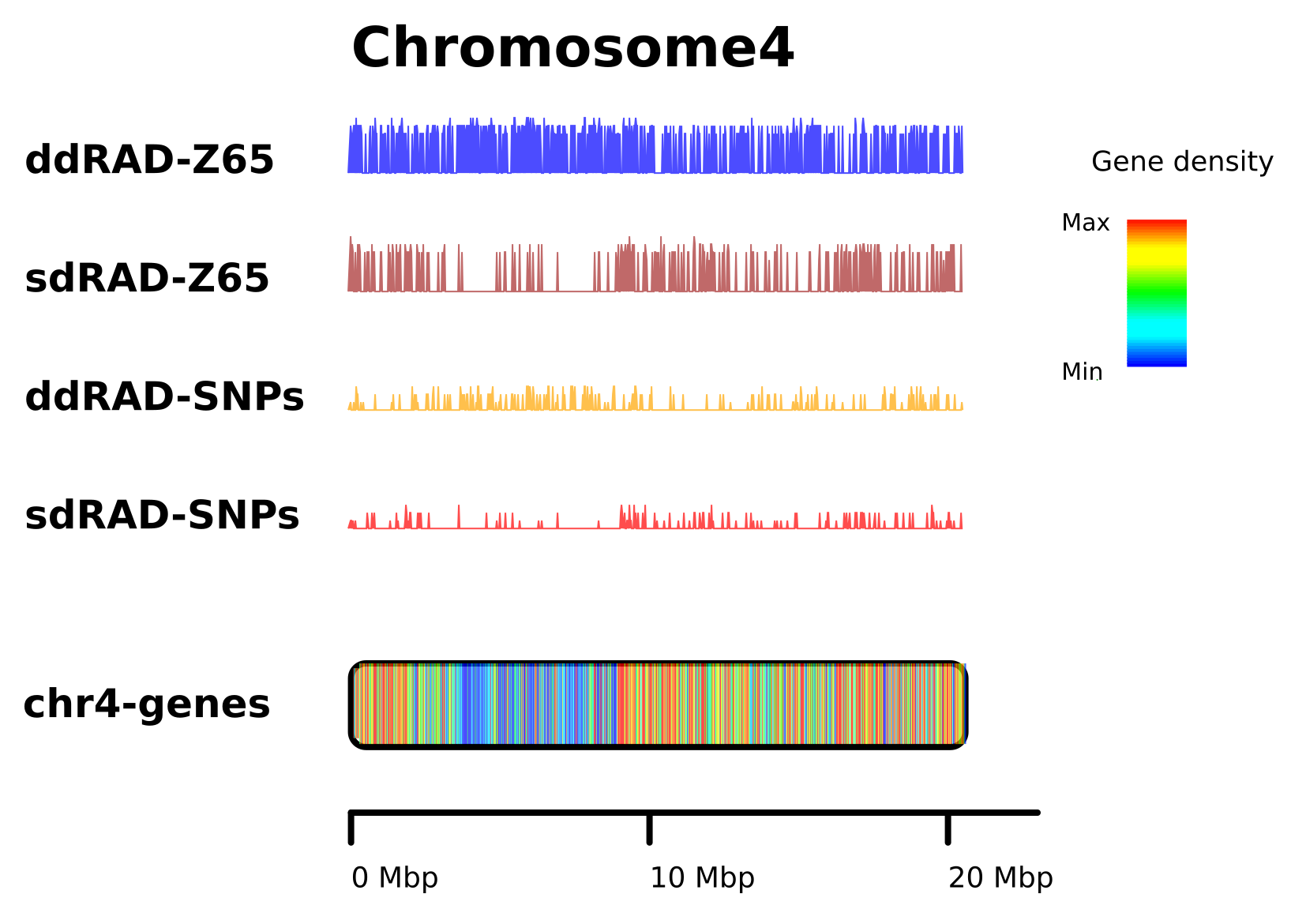

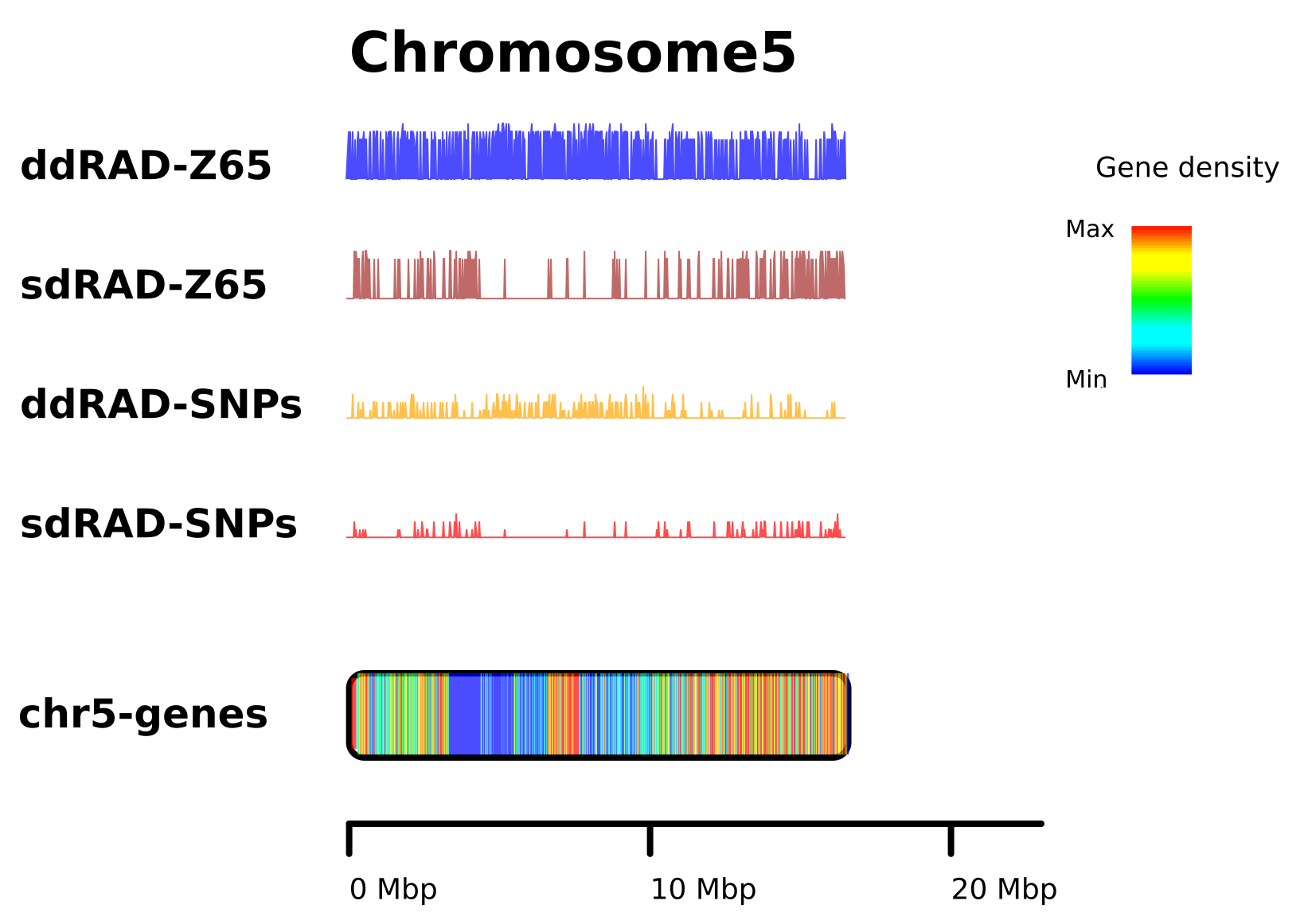

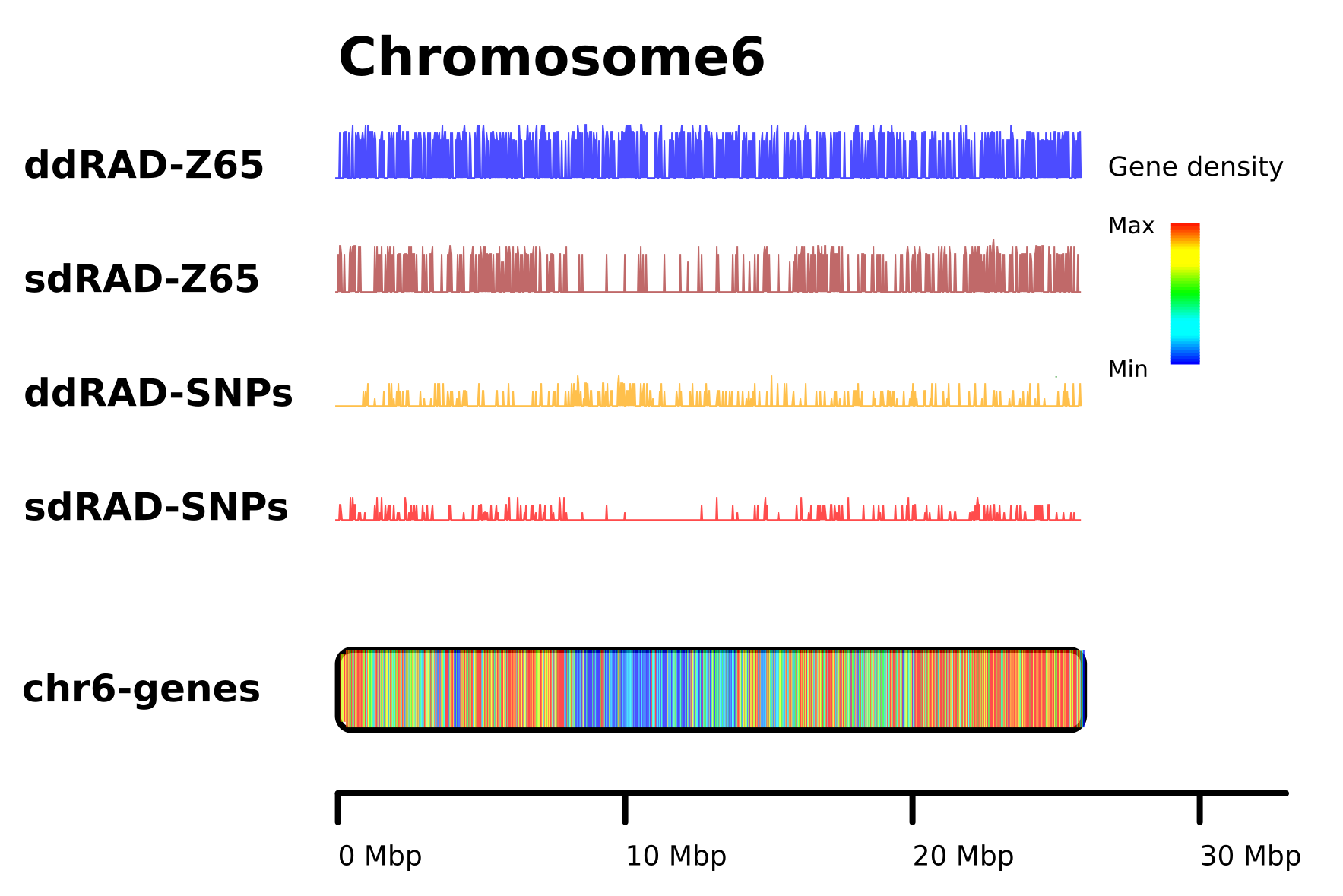

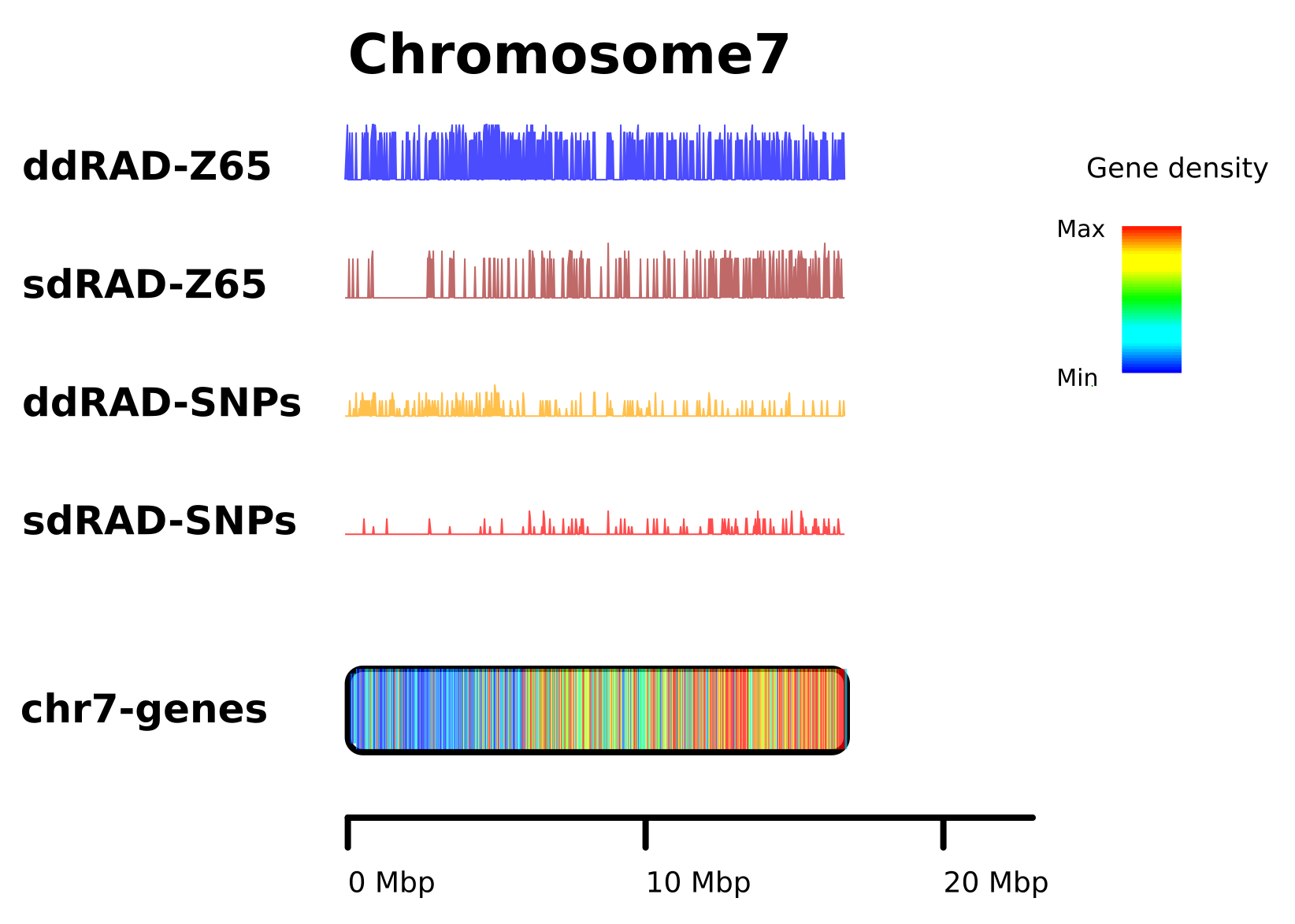

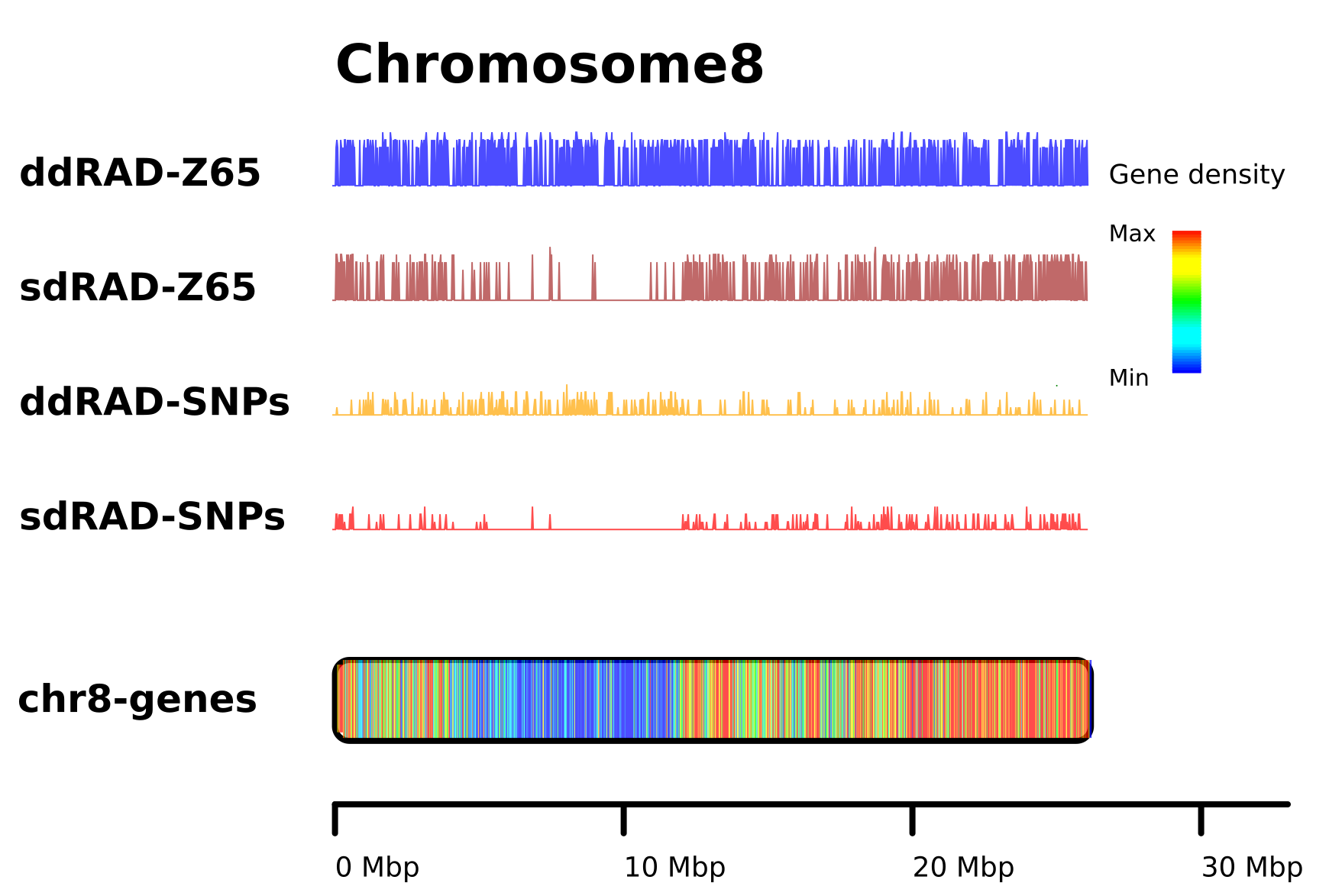

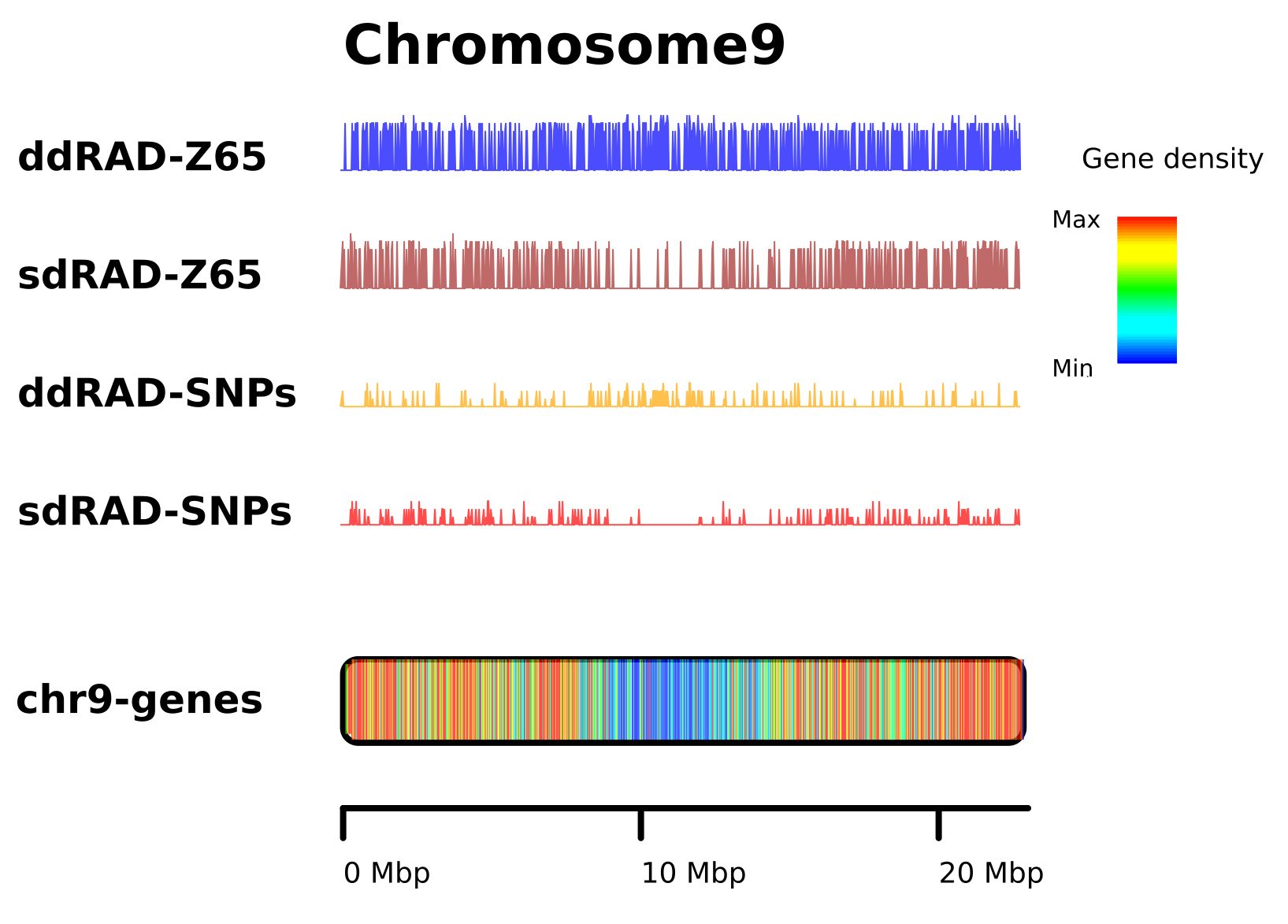

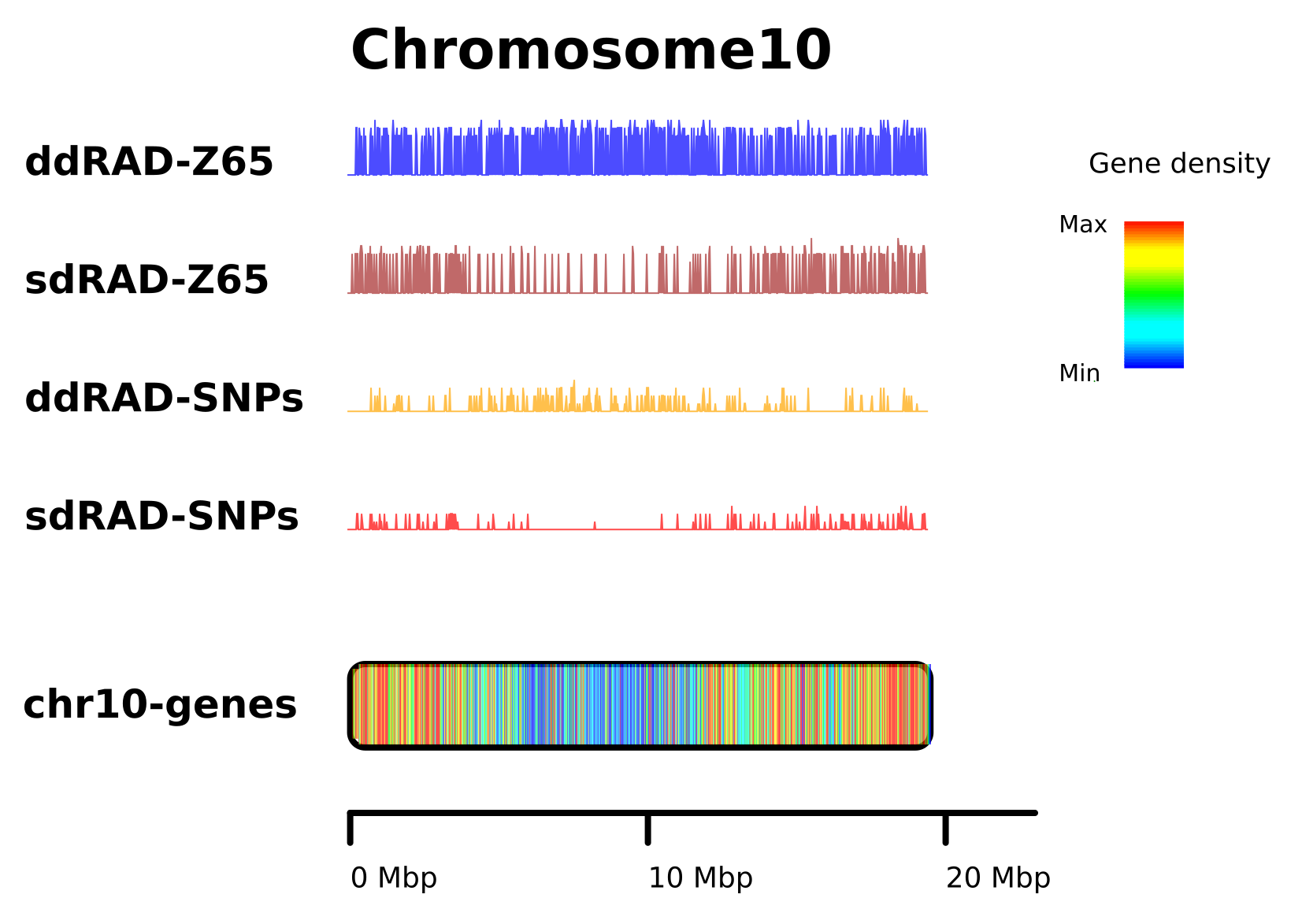

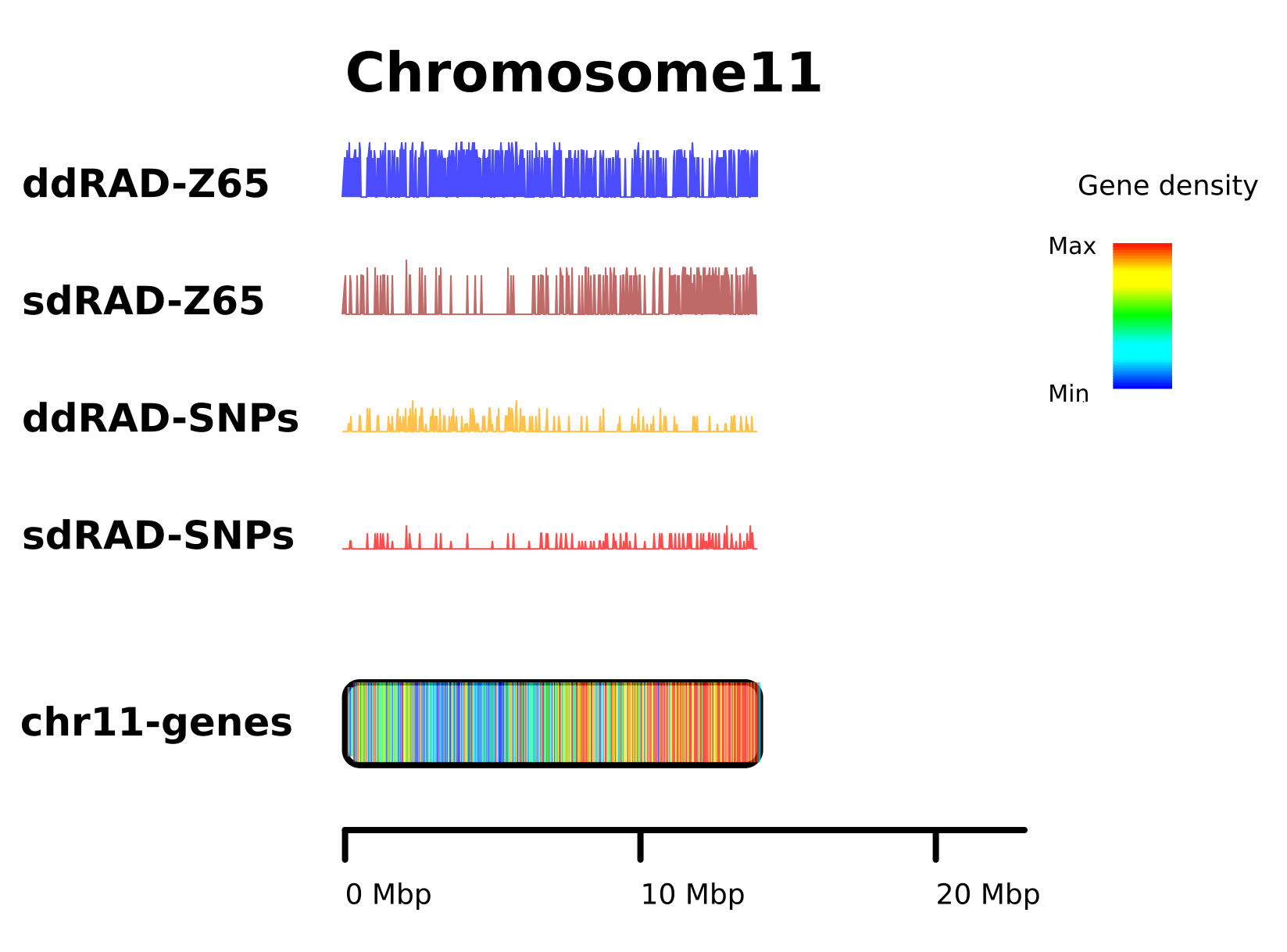

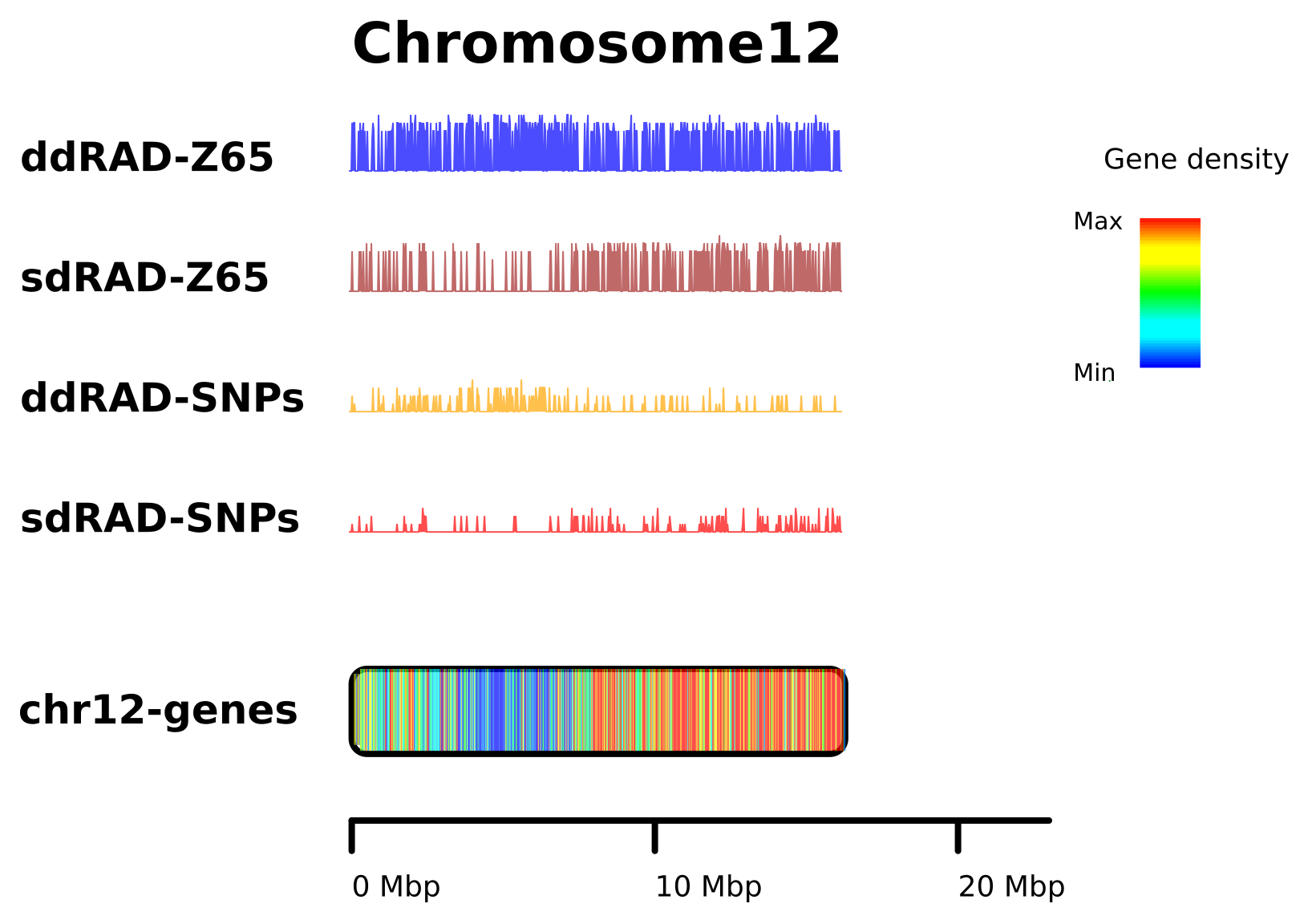

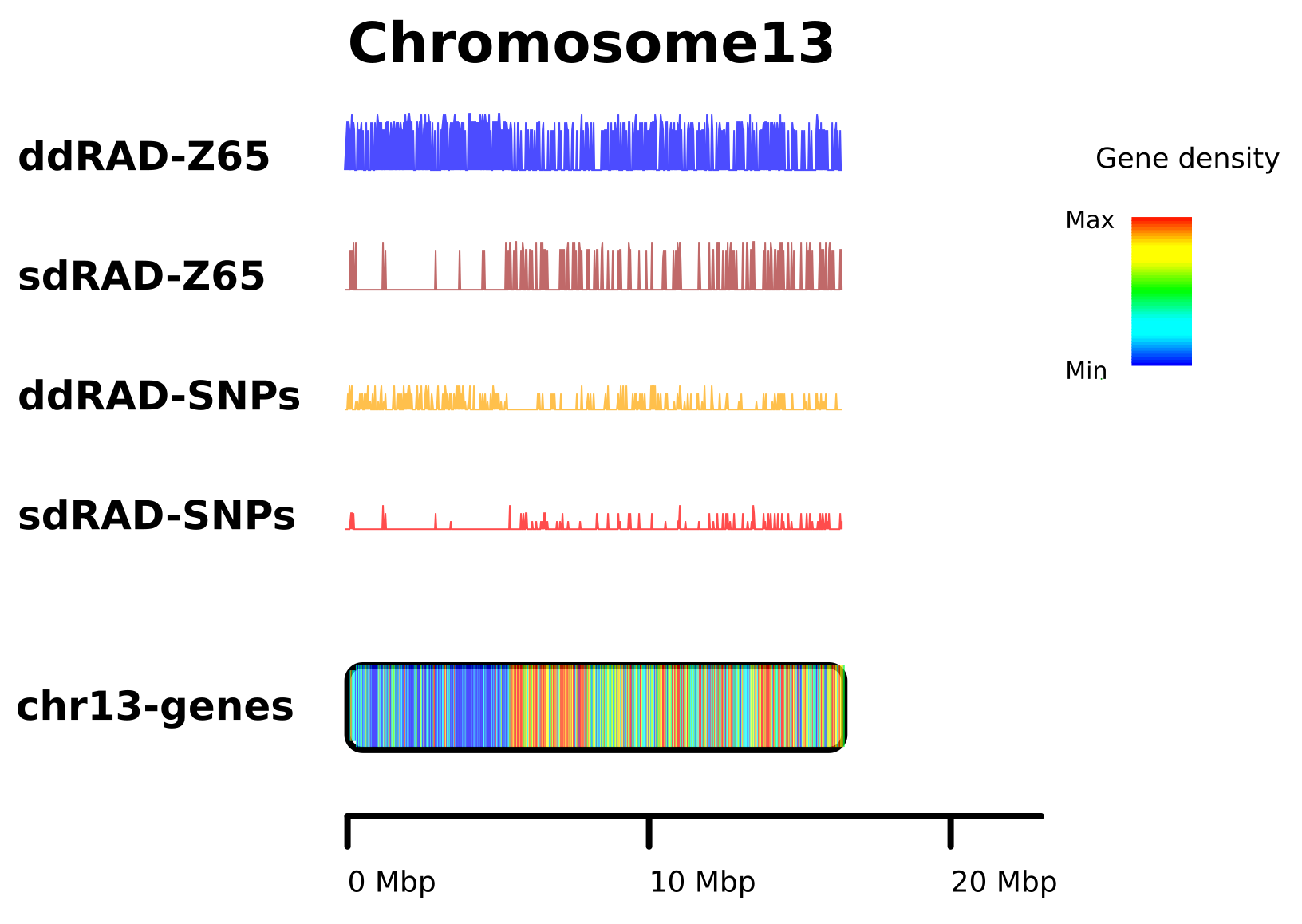


**Supplementary Figure 2:** A chromosome-wide distribution of ddRAD-seq and sdRAD-seq read mapping for Z65 sample, SNP density from both ddRAD-seq and sdRAD-seq datasets and gene density in heatmap format for all 13 chromosomes.
