# Supplementary material for: A pilot-scale comparison between single and double-digest RAD markers generated using GBS strategy in sesame (Sesamum indicum L.)
Source: PLoS One. 2023 Jun 2;18(6):e0286599. doi: 10.1371/journal.pone.0286599 (PMC10237379; doi:10.1371/journal.pone.0286599)
Supplement: Attachment — Submitted filename: Response to reviewer comment_R1.docx [file pone.0286599.s004.docx]

**Response to reviewer comments:**
**Reviewer #1:**

Line 435. How many base pair was used for size selection.

**Response:** size selection was 250-400 bp and mentioned in line 459 of revised manuscript.

Line 440. why you were select these enzymes. Do you have any pre-experiment for efficiency of enzymes.

**Response:** Based on the literature search and is cited now in the revised manuscript (line #465).

Line 442. Size selection. Length of size???

**Response:** Same protocol was used for both sdRAD and ddRAD hence 350-450 bp.

Line 444. What is your length of product. 150 x2? and single or paired?

**Response:** The read length is 2x150 bp (paired-end data) for both sdRAD and ddRAD. Detailed information is provided in Supplementary Table 1.

Line 447. What is your parameters in trimmomatic.

**Response:** The parameter details were described in the revised manuscript (lines 472-474).

Line 465. ….genes as described in (Ruperao et al., 2021). Missing sentence???

**Response:** The sentence is reframed in the revised manuscript (Line #492).

Line 175…..and ddRAD-seq datasets, respectively

**Response:** Revised accordingly in this version (line #175).

Line 272-273. Please add related references

**Response:** The part of manuscript pertaining to core development has been deleted throughout the manuscript, as suggested by the reviewer, and is reflected in track changes mode.

Line 383-392. There is one study has been conducted sesame for diversity analysis with the use of ddRAD-Seq approach. You should discuss the study
Basak, M., Uzun, B., and Yol, E. (2019). Genetic diversity and population structure of the mediterranean sesame core collection with use of genome-wide SNPs developed by double digest RAD-Seq. PLoS one 14:e0223757. doi: 10.1371/journal.pone.0223757

**Response:** The is updated in the revised manuscript (Line nos: 297-300 and 391-393).

Line 396. Identifying 8 samples as a core collection is not scientific approach. It is not suitable for core collection idea. Please delete all about core collection. Discussion is weak. Please discuss these methods advantages and disadvantages compared to other sequencing approach.

**Response:** The segment detailing the core collection was deleted and the text on the sequencing approaches for marker discovery has been included in the Discussion (Lines 283-302).

**Reviewer #2:**

**Some major comments:**
My first point concern the absence consideration of the fact that rad-sequencing (sd or dd) is highly dependent on the coverage of each samples. This mean that samples with less coverage will de-facto have information, either because some locus were not sequenced, or because the coverage in some locus might not be sufficient to call SNPs.
In the actual manuscript, this aspect is not discuss while it might have a huge impact on the diversity parameter inferred, either with the kmer approach or with the SNPs calling part.

**Response:** We agree that the sequence coverage for each sample with each RAD protocol varies and also it has an impact on the SNP calling. In our analysis, we have used appropriate filters to exclude low-frequency or missing data SNPs. In spite of the missing information, this method is a reduced-representation approach being widely used since it helps us to obtain a good number of SNPs (say in 1000s) with lesser genome coverage and at minimal cost. Only those loci that satisfies the set filters were carried forward for the downstream analysis.

- The introduction claims that genetic information exists for the species, but do not list them (at least if information about populations exist), nor discuss the results of this study in perspectives with previous experiments.

**Response:** The existing genetic information in the sesame were included in the discussion session of the revised manuscript (Lines 283-300).

- The choice of the enzymes, that will have a great impact on the number of locus is not explained nor discussed.

**Response:** The choice of the enzyme was based on the earlier report and cited now in the revised manuscript (Line #465).

- The choice of the strains considered in this study, what is know about their history and how the are related is absent in the actual document and does not allow to grasp the pertinence of the study.

**Response:** Based on our previous field experiments, we have identified the phenotypically diverse accessions and were used in this study. Further details were provided in the supplementary table 1.

- The choice of the methods chose to “evaluate the genetic diversity” is not explained. Why use a Kmer approach ? How does the number of reads per samples affect the results of this approach?

**Response**: K-mer based approach was chosen because it helps exclude the data with sequencing errors. Also, this approach provides computational efficiency. The diverse and genetically distant genotypes are expected to have more unique k-mers, more variant alleles and the possibility of higher heterozygosity. Number of reads when increased will be reflected in the depth of coverage and will help distinguish the heterozygosity vs SNPs with higher confidence.

- The description of the methods used does not allow the reproducibility of the experiment (Few examples : authors did not describe the size of the fragments selected, nor the parameters for trimming in trimomatic, nor the ones for SNP calling in STACKS)

**Response**: The revised manuscript (Lines #459, 465, 472-474) addresses the concern raised by the reviewer.

- Data are not made available, or at least, this is not stated in the manuscript.

**Response:** The raw data has been submitted to the public database and is accessible through PRJEB60972. The same is detailed in the revised manuscript, lines 518-520.

-Multiple statement through the document needs references.

**Response:** The references for the statements were suitably cited in the revised manuscript. In case the reviewer feels any specific portion that requires citation, we are happy to do it.

Some minor comments :
- Check reference line 431

**Response:** Reference included in the revised manuscript.

- Check tense through the document (example line 64: “were” is used wile present tense is used in the rest of the paragraph)

**Response:** Corrected in the revised manuscript.

*******
