# Supplementary material for: A pilot-scale comparison between single and double-digest RAD markers generated using GBS strategy in sesame (Sesamum indicum L.)
Source: PLoS One. 2023 Jun 2;18(6):e0286599. doi: 10.1371/journal.pone.0286599 (PMC10237379; doi:10.1371/journal.pone.0286599)
Supplement: Attachment — Submitted filename: Response to reviewer comment_R3.docx [file pone.0286599.s005.docx]

**Response to reviewer comments:**
**Reviewer #1:**

All comments have been addressed.

**Reviewer #2:**

**Some side comments:**
I would like to point that the version with the tracked change does not track all changes in the manuscript, making the review process harder. Please consider for future submission kipping track of all the modifications.

Also, number of lines pointing to changes does not match the actual changes (i.e “The existing genetic information in the sesame were included in the discussion session of the revised manuscript (Lines 283-300)”, changes are in lines 303 and onward..

**Response:** Apologies for the minor changes in line numbers that had happened due to last minute changes in the manuscript after drafting the response to reviewer document and got slipped to re-edit with correct line numbers. In the present revised manuscript, this is duly taken care of. Thanks for pointing out.

**Major comments:**

In their revised version and answer to comments, the authors answered some of my concerns but some caveats remains.

In my previous review, I was pointing that rad-sequencing (sd or dd) is highly dependent on the coverage of each samples, thus impacting the diversity parameters.

I do understand that the authors used defaults parameters in Stacks, but showing as a supplementary material that the coverage / total number of bp sequenced does not corelates with diversity statics could be useful for the reader to trust your results.

I also suggest to the authors to discuss this issue in the discussion.

**Response:** The coverage of mapped reads was assessed in both vertical coverage (read depth) and horizontal coverage (number of bases captured with sequence reads). The vertical coverage indicates the confidence of the genotype called (represents the quality of called genotype). The horizontal coverage represents the proportion of genome sequence covered (which represents how dense the variants were called, and at the downstream, diversity will be assessed). This is detailed in the discussion in revised manuscript between the line numbers 322-332.

I pointed in my first review that the authors does not describe the accessions used un this study. Indeed, authors provide in Sup Tab 1 the location of origin of the accession, but the link between them remains elusive to me. I still think the manuscript would benefit of a brief description of the reasons of inclusion of this list of accessions.

**Response:** In the revised manuscript (lines 102-104), basis of selecting the genotypes was mentioned. Also, in the ‘Supplementary Table 1’ submitted, the required phenotype trait information on basis of which the genotypes were selected was provided. This is now available for readers to have the background information on the choice of the list of accessions used in our study.

I also think that the manuscript contains languages error (grammar, syntax, vocabulary). I suggest the authors takes the time to thoroughly edit the manuscript.

**Response:** The revised manuscript was edited for correcting the language error (especially, in the Lines 24, 28, 48, 55, 65, 183, 244, 279-281, 346, 366, 367, 369, 386, 448, 468, 494-496, 503, and 506).

*******
